# Electrophysiological and olfactometric evaluation of long‐term COVID‐19

**DOI:** 10.14814/phy2.14992

**Published:** 2021-09-18

**Authors:** Andrea Mazzatenta, Claudia Montagnini, Andrea Brasacchio, Ferdinando Sartucci, Giampiero Neri

**Affiliations:** ^1^ Neuroscience, Imaging and Clinical Sciences Department ‘G. d’Annunzio’ Univeristy of Chieti‐Pescara Chieti Italy; ^2^ Centro di Selezione e Reclutamento Nazionale dell'Esercito Foligno (Pg) Italy; ^3^ U.O.C. Anestesia e Rianimazione Policlinico Militare ‘Celio’ Esercito Italiano Rome Italy; ^4^ Neurophysiopathology Unit Department of Clinical and Experimental Medicine Pisa University Medical School Pisa Italy; ^5^ Neuroscience Institute CNR Pisa Italy; ^6^ Don Carlo Gnocchi Foundation Marina di Massa, Massa Italy

**Keywords:** anosmia, long‐term COVID‐19, OERP, olfactometric evaluation, VOCs

## Abstract

COVID‐19 is a public health emergency with cases increasing globally. Its clinical manifestations range from asymptomatic and acute respiratory disease to multiple organ dysfunction syndromes and effects of COVID‐19 in the long term. Interestingly, regardless of variant, all COVID‐19 share impairment of the sense of smell and taste. We would like to report, as far as we know, the first comprehensive neurophysiological evaluation of the long‐term effects of SARS‐CoV‐2 on the olfactory system with potential‐related neurological damage. The case report concerns a military doctor, with a monitored health history, infected in April 2020 by the first wave of the epidemic expansion while on military duty in Codogno (Milan). In this subject, we find the electrophysiological signal in the periphery, while its correlate is absent in the olfactory bulb region than in whole brain recordings. In agreement with this result is the lack of metabolic signs of brain activation under olfactory stimulation. Consequently, quantitative and qualitative diagnoses of anosmia were made by means of olfactometric tests. We strongly suggest a comprehensive series of olfactometric tests from the first sign of COVID‐19 and subsequent patient assessments. In conclusion, electrophysiological and metabolic tests of olfactory function have made it possible to study the long‐term effects and the establishment of neurological consequences.

## Graphical abstract

(1) Olfactory impairment in long‐term COVID‐19. (2) Electrophysiological and olfactometric evaluation of long‐term COVID‐19. (3) COVID‐19 and neurological damage.

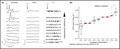

## 1 INTRODUCTION

The pandemic spread of SARS‐CoV‐2, which is transmitted through infected droplets emitted by breath, produces COVID‐19 which has a wide range of clinical manifestations ranging from asymptomatic and respiratory failure requiring support in an intensive care unit to multiple organ dysfunction syndromes (Cascella et al., 2020).

The main characteristic effect of the infection, irrespective of variant, is the aggression on the olfactory system with consequent impairment of the sense of smell and associate taste perception (Lechien, Chiesa‐Estomba, Hans, et al., 2020; Mazzatenta et al., 2020; Sungnak et al., 2020). This phenomenon has been associated with putative neurotropism of the virus as occurring in other human coronaviruses (for review see Cheng et al., 2020). Interestingly, the viral targets ACE2 and TMPRSS2 show extremely high expression in the characteristic cells of the nasal epithelium, goblet cells, and hair cells. Consequently, these cells are candidates as sites of original viral infection and possible reservoirs of dissemination, and SARS‐CoV‐2 is an enveloped virus that does not require cell lysis for viral release. Thus, the virus could exploit existing secretory pathways in nasal goblet cells for continuous low‐level release in the early phase without obvious pathology (Sungnak et al., 2020). In preliminary observations, obstruction of the olfactory fissure has been evaluated as a factor involved in increasing disease severity (Lechien, Chiesa‐Estomba, Place, et al., 2020). A key element is understanding the pathophysiological mechanisms underlying the impairment of smell and taste in COVID‐19, including potential viral spread through the olfactory neuroepithelium and invasion of the olfactory bulb and central nervous system (Lechien, Chiesa‐Estomba, Hans, et al., 2020).

Consequently, the long‐term effect of COVID‐19 suggests a large public health emergency because it affects millions of cases globally. We report a case of anosmia in a long‐term post‐COVID‐19 patient without hospitalization, examined with olfactory smart threshold test (OST test) and electrophysiological techniques: olfactory event‐related potential (OERP) and olfactory real‐time volatile organic compound test (ORT‐VOC test).

## 2 CASE

A 34‐year‐old male patient was evaluated by our laboratory on June 14, 2021 because reporting symptoms of anosmia, ageusia coupled with cacosmia, referred to a burnt and musty smell; he also reports mild dyspnea, muscle fatigue, and tiredness. The patient clinically appears to be healthy and not currently taking any medication; vital signs and physical examination were as follows: blood pressure max 125.8 mmHg ± 2.89 SD and min 76.6 mmHg ± 3.27 SD; pulse rate 63.1 times/min ± 1.85 SD; respiratory rate 16 times/min ± 1.49 SD; temperature 36.35℃ ± 0.05 SD; and SpO_2_ 97.8% ± 0.63 SD on room air. Chest x‐ray showed bilateral apical opacities on the lungs and brain CT scan showed no alterations. Laboratory tests and blood gas analysis do not reveal any abnormalities.

The patient has a complete health history and is a military doctor who was deployed to Codogno (Milan, Italy) for the first COVID‐19 emergency in March 2020 and was infected with SARS‐CoV‐2. Following symptoms of anosmia, ageusia, fever, severe asthenia, muscle pain, disorientation, mild amnesia, and dyspnea, the patient underwent a molecular swab test on April 4, 2020, which was positive. The patient remained positive for approximately 30 days and the first negative swab was on May 5, 2020.

**FIGURE 1:**
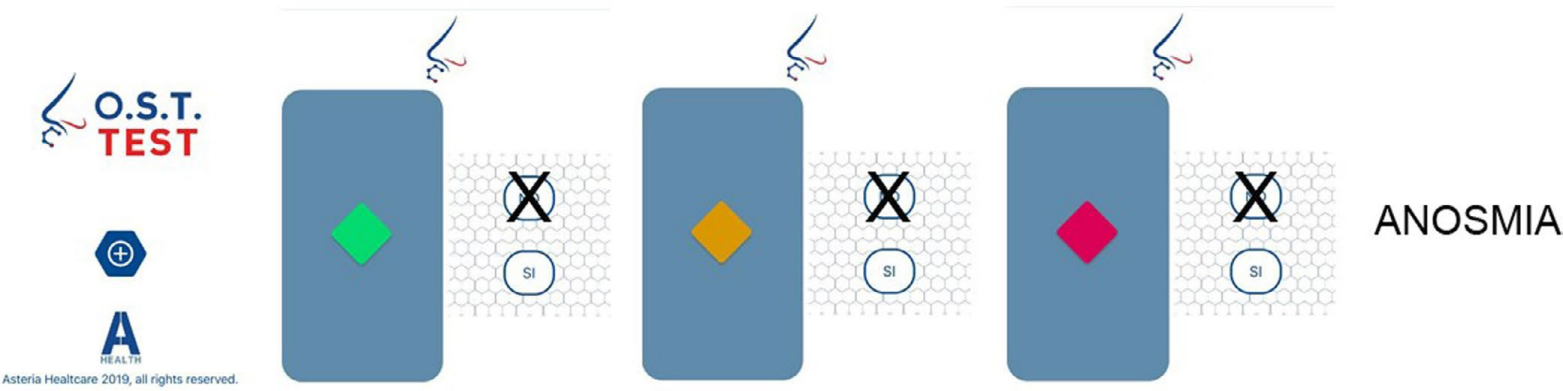
OST test results. The patient does not perceive any of the three stimulations of the test with exponentially increasing concentration and is, therefore, assessed as anosmic. OST, olfactory smart threshold

A preliminary OST test (validated in COVID‐19 by Mazzatenta et al., 2020) was carried out and the results are shown in Figure 1. The OST test is useful for preliminary fast screening of the olfactory function and it works based on four disposable items based on Connecticut Chemosensory Clinical Research Center (C.C.C.R.C.) threshold test (Cain et al., 1988) and Italian population age phenotype threshold test (Mazzatenta et al., 2016). The OST test consists of a logarithmic scale of increasing concentrations of n‐butanol. The test involves answering “YES” or “NO” to the presentation of three coloured ampoules in the following order green, yellow and red indicating the three increasing concentrations of *n*‐butanol, and a white odourless ampoule which is the negative control test presented preliminarily. The affirmative answer to the green vial is considered normosmia, i.e. the subject has a normal olfactory threshold value; if the answer is negative, the subject moves on to the next colour. The affirmative answer to the yellow vial corresponds to an evaluation of hyposmia, i.e. an alteration of the olfactory threshold, if the answer is negative, the subject moves on to the red vial. If it answers positively it is severe hyposmia, that is an important alteration of the olfactory threshold, if it answers “NO” then it corresponds to an evaluation of the olfactory threshold of anosmia (Mazzatenta et al., 2020).

**FIGURE 2:**
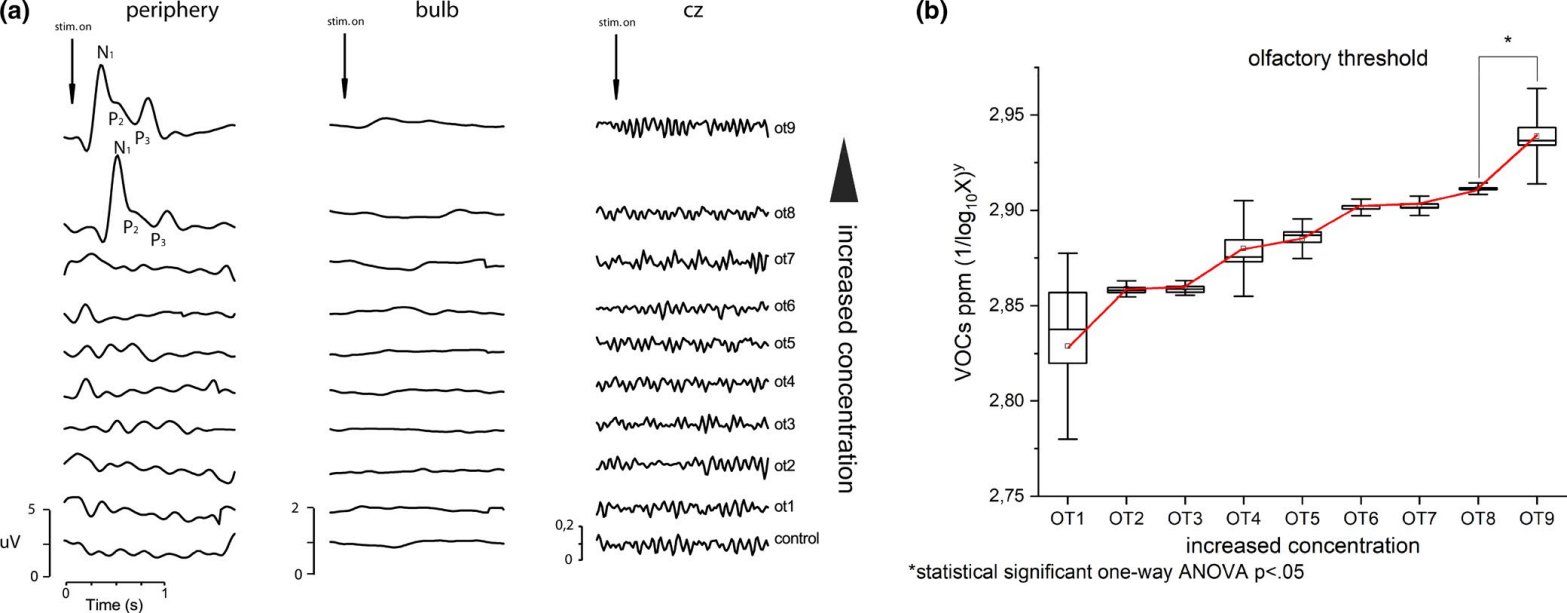
Results of electrophysiological recordings of the modified Cain test. (a) Olfactory event‐related potential and (b) VOCs exhaled response to increasing *n*‐butanol concentration. VOCs, volatile organic compound

In Figure 2, the results of the olfactory threshold investigated using a modified Cain test (Cain, 1982; Cain et al., 1983) coupled with electrophysiological recordings are shown. OERPs are an international validated electrophysiological technique for the study of the olfactory system (Lötsch & Hummel, 2006). This assessment is an objective method to observe changes in olfactory function independent from patients’ response bias. The presence of OERP is a robust indicator of a healthy olfactory function; conversely, the absence of OERP suggests an olfactory loss (Lötsch & Hummel, 2006). Because, the transmission of olfactory sensory input travels from the olfactory neuroepithelium located into the nasal cavities toward the olfactory bulbs through the first cranial nerves, which here makes contact with the second‐order neurons the mitral and tufted cells within glomeruli. From here, the postsynaptic fibers that form the olfactory tracts project to the primary olfactory areas, which comprise the anterior olfactory nucleus, tenia tecta, olfactory tubercle, piriform cortex, amygdale, anterior cortical amygdaloid nucleus, periamygdaloid, and entorhinal cortices. The piriform cortex is connected to thalamus, hypothalamus, and orbitofrontal cortex, and the entorhinal cortex is connected to hippocampus. The thalamus has connections toward secondary olfactory areas, as the OFC and insular cortex (Giessel & Datta, 2014). Consequently, OERPs are the result of sequential activation of different brain areas that begins from periphery and involves a number of brain areas. Typically, OERPs consist of a negative component, the N_1_, followed by two positive components, P_2_ and P_3_. The early components reflect the exogenous cortical activity related to sensory input detection and primary sensory processing, while the later OERP components reflect endogenous cortical activity related to secondary cognitive processing (Lötsch & Hummel, 2006). Latency (range from 530 to 800 ms after stimulus onset), amplitude (approximately between 4 and 20 µv), and shape are the main parameters of OERP components (Lötsch & Hummel, 2006). OERPs are the results of a grand average of 10 stimulations, recorded by a EEG power lab equipment's (AD‐Instruments) following standard procedure (Invitto & Mazzatenta, 2019; Lötsch & Hummel, 2006; Tateyama et al., 1998; Wang, Chen, et al., 2004; Wang, Hari, et al., 2004) and are only detectable in the periphery at high concentrations (Figure 2a). Exhaled VOCs’ response to exponential growing concentration of *n*‐butanol was recorded by the ORT‐VOC test using an e‐nose (iAQ‐2000; Applied Sensor), (Cain, 1982, Cain et al., 1983). The ORT‐VOC test is based on the changes in metabolites exhaled during sensory stimulation compared to the unstimulated basal recording (Mazzatenta, Pokorski, & Di Giulio, 2015; Mazzatenta, Pokorski, Montinaro, et al., 2015; Mazzatenta, Pokorski, Sartucci, et al., 2015; Mazzatenta et al., 2016). The ORT‐VOC test results in a slightly significant response at the highest concentration of *n*‐butanol (OT9) (Figure 2b). Convergent results point to a diagnosis of quantitative anosmia.

**FIGURE 3:**
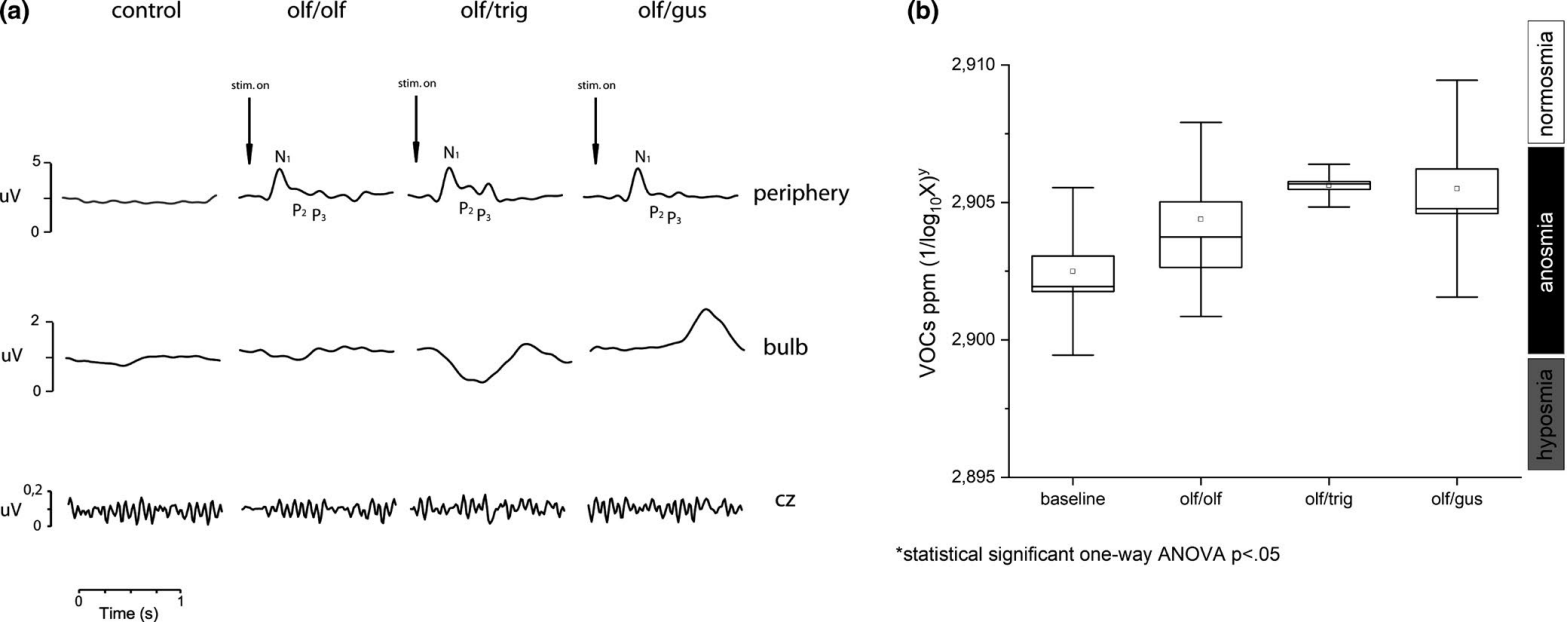
Results of electrophysiological recordings of olfacto/olfactive, olfacto/trigeminal, and olfacto/gustative responses. (a) Olfactory event‐related potential and (b) VOCs exhaled response to olfacto/olfactive, olfacto/trigeminal, and olfacto/gustative stimulations. VOCs, volatile organic compound

Furthermore, olfactometric investigations were the evaluation of olfacto/olfactive, olfacto/trigeminal, and olfacto/gustatory responses with electrophysiological recordings (Figure 3). OERPs for the three stimulations are only present peripherally (Figure 3b). No significant change for the three stimulations is for the exhaled VOCs compared to the unstimulated baseline (Figure 3a).

Convergent results point to a diagnosis of qualitative anosmia.

Finally, taste stimulation with suprathreshold 0.5 g/mL of sucrose, and 0.5 g/mL of sodium chloride, 0.5% of citric acid, and quinine provided a positive response.

## 3 DISCUSSION

With the rapid increase in the number of COVID‐19 patients and the appearance of various symptoms and signs, reports of neurological damage have gradually attracted attention (Chiappelli, 2020; Pezzini & Padovani, 2020).

COVID‐19 neurological early disorders, anosmia and ageusia, are of great interest because SARS‐CoV‐2 was initially thought to have great difficulty passing through the blood–brain barrier (BBB), but postmortem investigations of patients and the use of advanced models of the human BBB have shown: the spike protein (S) binding receptor of SARS‐CoV‐2, ACE2, is widely expressed in brain microvascular endothelial cells; the S protein can directly damage the integrity of the BBB to varying degrees; the S protein can induce the inflammatory response of microvascular endothelial cells that change the function of the BBB (Pezzini & Padovani, 2020; Tsai et al., 2005). These findings support the hypothesis that SARS‐CoV‐2 can potentially enter the brain via the olfactory route, alter the BBB and enter the brain, leading to neurological manifestations, with the possibility of producing encephalitis (Pezzini & Padovani, 2020; Tsai et al., 2005).

In the reported case, there is evidence of potential neurological damage, at least in the olfactory system. This is evidenced by the presence of peripheral signals in the olfactory sensory epithelium lining the turbinates, suggesting that olfactory sensory neurons (OSNs) after initial infection have been renewed; in fact normal regeneration occurs in about 30 days. In contrast, no signal was detected in the main olfactory bulb or in the brain for both the threshold and the three qualitative olfactory stimulations. In accordance with studies carried out using OERPs on neurodegenerative forms (Invitto et al., 2018). Similarly, VOC analysis did not reveal metabolic changes induced by olfactory stimulation. Consequently, the diagnosis is quantitative and qualitative anosmia, in agreement with previous studies (Mazzatenta, Pokorski, & Di Giulio, 2015; Mazzatenta, Pokorski, Montinaro, et al., 2015; Mazzatenta et al., 2016). Taste is preserved confirming that the impairment is limited to the olfactory pathway, in agreement with previous studies (Mazzatenta et al., 2020).

This could be interpreted as a disruption somehow along the neuronal pathway from the cribriform plate to the olfactory circuitry in the brain, which could reflect a potential viral neuroinvasion of the CNS with lesions occurring, at least, in the cellular pathway of the olfactory system. The question that arises is whether SARS‐CoV‐2 extinguishes its effect by disrupting somehow the olfactory cellular pathway or could progress into the CNS.

As far as we know, this is the first comprehensive neurophysiological evaluation of the effects of SARS‐CoV‐2 on the olfactory system showing clear neurological damage suggesting that the patient should be assessed with a full set of olfactometric tests. The early effects on the olfactory system are a putative indication, found naturally in a single patient and to be confirmed by studies on larger series, of the neurological changes induced by virosis and support the potential basis for long‐term neurological sequelae.

## CONFLICT OF INTEREST

All authors have nothing to disclose.

## AUTHOR CONTRIBUTION

A.M.: Conceptualization, methodology, data curation, formal analysis, figure preparation, writing original draft preparation; C.M., A.B., and G.N.: medical inspection; A.M., C.M., and G.N.: validation; G.N.: editing and supervision.

## ETHICAL STATEMENT

Ethical clearance protocol number colf01.2020 from “Comitato Etico delle Province di Chieti e di Pescara” has been obtained.

## Data Availability

Data available in a public institutional repository.
